# Longitudinal Outcomes of Oral Glutamatergic Augmentation in Trauma-Related Dissociation: A Six-Month Case Study

**DOI:** 10.7759/cureus.108751

**Published:** 2026-05-12

**Authors:** Ngo Cheung

**Affiliations:** 1 Psychiatry, Cheung Ngo Medical Limited, Hong Kong, HKG

**Keywords:** cheung regimen, dissociative fugue, dxm, glutamatergic, ptsd, trauma

## Abstract

Trauma-related dissociation can present with derealisation, depersonalisation, abrupt lapses in awareness, memory gaps, and fugue-like states, and it may be difficult to treat with standard approaches alone. Glutamatergic interventions have been proposed as neuroplasticity-focused strategies because N-methyl-D-aspartate (NMDA) receptor antagonism and downstream alpha-amino-3-hydroxy-5-methyl-4-isoxazolepropionic acid (AMPA)-linked signalling may influence synaptic plasticity.

A 25-year-old woman with documented complicated grief related to her paternal grandfather’s sudden death, longstanding derealisation, recurrent blank-outs, and fugue-like episodes presented with moderate depressive symptoms and moderate-to-severe anxiety. Over approximately seven months of documented outpatient psychiatric follow-up from 14 October 2025 to 4 May 2026, she was treated in an outpatient setting with an oral regimen consisting of dextromethorphan 30-60 mg/day, fluoxetine 10 mg/day, piracetam 600-1200 mg/day, and low-dose antipsychotic medication, with later medication adjustments according to tolerability and clinical response. Depressive symptoms improved gradually from moderate to mild severity. The most notable clinical change was the complete and sustained resolution of dissociative blank-outs and fugue-like states, accompanied by improved work performance, fewer unnoticed errors, and better self-monitoring. Generalized anxiety and relationship-triggered emotional reactivity improved only partially. Morning tiredness was reported early in treatment; however, the clinical record did not specify enough information to attribute this definitively to medication timing. This case suggests that oral NMDA-AMPA modulation may support progressive recovery of cognitive integration and functional capacity in trauma-related dissociation, while residual anxiety and interpersonal reactivity may require additional psychotherapeutic or targeted approaches. The case adds hypothesis-generating longitudinal support for low-cost glutamatergic augmentation in routine outpatient practice.

## Introduction

Dissociation refers to a disruption or discontinuity in the normal integration of consciousness, memory, identity, emotion, perception, body representation, motor control, or behaviour [1]. Clinically, dissociative symptoms may include depersonalisation, derealisation, amnesia, absorption, emotional numbing, identity alteration, or episodes of reduced responsiveness. Derealisation is the experience that the external world feels unreal, dreamlike, distant, foggy, or visually altered, whereas depersonalisation is the experience of detachment from one’s own body, thoughts, emotions, or sense of self [1]. Fugue-like states are used here descriptively to refer to episodes of abrupt disruption in awareness or autobiographical continuity, with subsequent memory gaps or apparently purposeful behaviour that is poorly recalled; the term is not used to imply that formal diagnostic criteria for dissociative fugue were established.

Post-traumatic stress disorder (PTSD) is a trauma- and stressor-related disorder that requires exposure to actual or threatened death, serious injury, or sexual violence, followed by symptoms from four major clusters: intrusion, avoidance, negative alterations in cognition and mood, and alterations in arousal and reactivity, with associated duration, impairment, and exclusion criteria [1]. The dissociative subtype of PTSD requires that full PTSD criteria are met and that the individual also experiences persistent or recurrent depersonalisation, derealisation, or both [1-4]. Around one-quarter to one-third of people with PTSD have been reported to show prominent dissociative features, especially derealisation and depersonalisation [2,3]. These patients may respond less predictably to standard trauma-focused psychotherapies and monoaminergic medication than patients with non-dissociative PTSD, and many continue to experience functional impairment despite repeated treatment attempts [4-6].

Usual management of PTSD includes trauma-focused psychotherapies, such as trauma-focused cognitive behavioural therapy, cognitive processing therapy, prolonged exposure, and eye movement desensitisation and reprocessing, together with pharmacological options when psychotherapy is unavailable, declined, insufficient, or clinically unsuitable [5,6]. Recommended pharmacotherapies commonly include selective serotonin reuptake inhibitors and serotonin-noradrenaline reuptake inhibitors, particularly sertraline, paroxetine, and venlafaxine in major guidelines [5,6]. For dissociative PTSD or trauma-related dissociation, staged or phase-oriented approaches may be considered, with attention to stabilisation, grounding, emotion regulation, interpersonal safety, and careful pacing of trauma processing [4].

Persistent dissociation, fugue-like states, and fragmented cognitive integration suggest that some presentations may require treatment approaches that move beyond conventional fear-extinction and monoamine-based models. Glutamatergic pathways refer to neurotransmission mediated by glutamate, the major excitatory neurotransmitter in the central nervous system. N-methyl-D-aspartate (NMDA) and alpha-amino-3-hydroxy-5-methyl-4-isoxazolepropionic acid (AMPA) receptors are central to synaptic plasticity, learning, memory, and experience-dependent circuit modification. Oral glutamatergic augmentation, as used in this report, refers to the off-label clinical strategy of using orally administered agents that influence glutamatergic signalling, including NMDA-receptor antagonism and putative AMPA-linked downstream plasticity effects, as adjunctive treatment for trauma-related symptoms.

Recent genomic and transcriptomic work has reframed PTSD as a condition involving developmental vulnerabilities in synaptic organisation and white-matter integrity. Large-scale genome-wide association studies have identified enrichment in genes related to synaptic pruning, complement signalling, particularly C4A, major histocompatibility complex pathways, and oligodendrocyte maturation [7]. These findings support a proposed model, rather than an established clinical doctrine, in which genetically and environmentally influenced mis-pruning during sensitive developmental periods produces immature corticolimbic and myelin-related circuits. Later trauma may then overwhelm this vulnerable structure, leading to plasticity failure and subsequent symptom consolidation through senescence-related processes [7,8]. This “pruning-vulnerability cascade” is a theoretical framework that provides a possible explanation for why some people develop chronic dissociation and cognitive fragmentation after early adversity, instead of showing only exaggerated fear responses in adulthood.

Treatments that temporarily enhance neuroplasticity have attracted attention because they may act on these circuit-level disturbances. Intravenous ketamine has been shown to reduce PTSD symptoms rapidly by blocking NMDA receptors, triggering an AMPA-mediated glutamate surge, and activating downstream brain-derived neurotrophic factor (BDNF) and mammalian target of rapamycin (mTOR) signalling involved in synaptogenesis [9,10]. However, ketamine treatment remains limited by cost, clinic infrastructure, and the need for supervised administration. Oral agents that engage overlapping glutamatergic pathways may offer a more accessible option. Dextromethorphan combined with cytochrome P450 2D6 (CYP2D6) inhibitors, such as fluoxetine or bupropion, may prolong dextromethorphan exposure, and AMPA-positive or neuroplasticity-associated modulators such as piracetam have been proposed as adjuncts to support downstream plasticity [11-14]. Open-label observations have described ketamine-like clinical effects using such regimens at lower cost and without infusion requirements [11,12]. These approaches may reopen plasticity windows in circuits affected by developmental and trauma-related dysregulation.

The hypothesised relevance to dissociation is distinct from a purely antidepressant effect. Mood improvement may reflect reduced depressive affect, whereas improvement in dissociation would be expected to involve restoration of continuity in awareness, memory, self-monitoring, and executive integration. This distinction is important because dissociative symptoms may improve, persist, or fluctuate independently of depression and anxiety scores.

Despite increasing interest in oral glutamatergic augmentation, most available reports describe short follow-up periods, often around six to eight weeks. There is limited longitudinal information on symptom specificity, medication scheduling, dose adjustment, tolerability, and functional outcomes after the early response phase. The present report addresses this gap by describing a longitudinal outpatient course in a young woman with longstanding trauma-related derealisation and fugue-like episodes. The case documents changes in symptoms, functioning, medication dose and frequency, and response across dissociative, depressive, and anxiety domains in routine outpatient care.

## Case presentation

Patient information and initial presentation

A 25-year-old woman was referred to an outpatient private psychiatric clinic in October 2025 because of persistent work difficulties and recurrent memory gaps (Table 1). She described a longstanding feeling that “nothing is real,” which she reported had been present since secondary school. At the time of presentation in 2025, this indicated a duration of several years since secondary school, although the exact year of onset was not documented. At work, she struggled to concentrate and made frequent careless mistakes, many of which she did not notice herself. These problems had become more visible after she moved into a new work environment. The exact duration since the work-environment change, the nature of the job, and the specific occupational setting were not documented.

**Table 1:** Longitudinal clinical course and pharmacotherapy summary over 28 weeks of follow-up - clinical assessment + = positive; - = negative; — = not assessed or not prescribed; ANX = anxiety screen result; DEP = depression screen result; GAD-7 = Generalised Anxiety Disorder 7-item scale; PHQ-9 = Patient Health Questionnaire-9 All medications were initially administered at night (QN). From 12 January 2026, dextromethorphan and piracetam were divided into twice-daily dosing. ^a^GAD-7 increased from 8 to 14 despite PHQ-9 improvement, indicating persistent broad anxiety re-emergence in the context of interpersonal stressors. ^b^No prescription list was included in the 9 March 2026 clinical note; no medication side effects were reported at that visit. ^c^PHQ-9 and GAD-7 scores were not documented on 25 April 2026.

Visit	PHQ-9 (0-27)	GAD-7 (0-21)	DEP	ANX	Key clinical findings
14 Oct 2025 (baseline)	14	16	+	+	Chronic derealisation since secondary school. Inability to concentrate at work; frequent careless mistakes with poor self-awareness. Episodic unresponsiveness and rumination with abrupt forgetting. History of self-talking, interpersonal sensitivity, and obsessive attachment in relationships. Recent breakup followed by heavy alcohol use approximately five days per week. Trauma-linked intrusive imagery and guilt related to paternal grandfather's death. Exact occupational role, episode duration, academic impact, family history, and detailed trauma history were not documented.
1 Dec 2025 (week 7)	12	11	+	+	Mood and work performance improved. Fewer dissociative episodes; fewer work mistakes. Morning tiredness noted. Mood affected by proximity to a local fire incident.
12 Jan 2026 (week 13)	9	8	−	+	More stable overall. No further blanking-out episodes. Sleep satisfactory. Increased appetite and occasional dullness reported.
9 Mar 2026 (week 21)	6	14^a^	−	+	PHQ-9 residual items: guilt/self-criticism (2), concentration difficulty (2), low energy (1), appetite change (1); all other items scored 0. GAD-7: all seven items scored 2. No medication side effects reported.^b^
25 Apr 2026 (week 28)	—^c^	—^c^	—	—	Mood stable. No further dissociative fugue. Work performance improved. Less appetite noted while taking aripiprazole; clinical valence and causality uncertain. Emotional reactivity triggered by relationship conflicts; as-needed benzodiazepine added.

She also described episodes in which she would suddenly become unresponsive during conversations and sometimes forget what had just been discussed. These episodes tended to occur after periods of intense rumination. The total duration in months or years of these episodes and the duration of each episode in minutes were not documented. Although attention-deficit/hyperactivity disorder was initially considered, the broader pattern of chronic derealisation, abrupt memory disruption, and sudden lapses in awareness was more consistent with dissociative phenomena.

Her psychosocial history was notable for trauma-related complicated grief. During her earlier life, her paternal grandfather had a prolonged illness. She recalled wishing that his suffering would end, and after he was later found to have died suddenly, she developed intense guilt, believing that she might have been able to help if she had found him earlier. These memories continued to return as vivid images associated with strong guilt. The grandfather’s specific illness, the duration of illness, whether he lived in the same home, the household composition, the exact age of the patient at the time, and the frequency of intrusive memories were not documented. It was also not documented whether derealisation began before or after the grandfather’s death. No other specific childhood-trauma details were documented in the available clinical notes.

She also reported unstable relationships marked by obsessive attachment. At presentation, she had recently ended one relationship and was involved in another that she described as difficult. The quality of family relationships, detailed relationship history, and any history of emotional, physical, or sexual abuse were not documented. She had also been drinking heavily, approximately five days per week, with episodes of becoming drunk easily. The number of years since alcohol use began, the amount consumed per occasion, and whether alcohol use continued or stopped after treatment began were not documented. Other substance use was not documented.

Past medical history, past psychiatric diagnoses, previous psychiatric hospitalisations, family psychiatric history, family medical history, academic history, social history, quality of friendships, baseline physical examination, neurological examination, vital signs, laboratory investigations, and radiological investigations were not documented in the available record. No baseline medication list before the October 2025 regimen was provided. A formal, structured mental status examination was not recorded, although the notes described dissociative unresponsiveness, rumination, self-talking in the past, interpersonal sensitivity, guilt, low mood, anxiety, and concentration difficulty.

Baseline screening showed a Patient Health Questionnaire-9 (PHQ-9) score of 14 and a Generalised Anxiety Disorder-7 (GAD-7) score of 16 [15,16], indicating moderate depressive symptoms and moderate-to-severe anxiety. The working clinical diagnosis was trauma-related dissociation with chronic derealisation and fugue-like/blanking-out episodes, accompanied by depressive and anxiety symptoms. Dissociative PTSD was considered clinically relevant but was not formally established because full Diagnostic and Statistical Manual of Mental Disorders, Fifth Edition, Text Revision (DSM-5-TR) PTSD criteria, including a structured Criterion A-E assessment and a validated dissociation scale, were not documented.

Initial regimen

After assessment, the oral regimen was started in October 2025 to target presumed synaptic dysregulation related to trauma history and dissociative symptoms (Table 2). The initial regimen consisted of dextromethorphan 30 mg/day, fluoxetine 10 mg/day to inhibit CYP2D6-mediated metabolism of dextromethorphan, piracetam 600 mg/day, and risperidone 0.5 mg/day. The prescription record documented once-daily dosing for these agents; the exact administration time was not documented in the available record.

**Table 2:** Pharmacotherapy regimen at each visit ★ = newly added agent; BID = twice daily; Δ = schedule change (same total daily dose); NR = not recorded; PRN = as needed; QD = once daily; QN = every night; ↑ = dose increase; — = not assessed or not prescribed The prescription record documented frequency but did not consistently document the exact time of day. Therefore, this table focuses on daily dose and frequency rather than assuming nightly administration. ^a^No prescription list was included in the 9 March 2026 clinical note. ^b^Daily dose increased from 30 mg/day to 60 mg/day. ^c^Total daily dose remained 1200 mg/day; schedule changed from once daily to twice daily. ^d^Patient reported not taking risperidone; it was no longer listed in the prescription set.

Medication	14 Oct 2025	1 Dec 2025	12 Jan 2026	9 Mar 2026	4 May 2026
Fluoxetine	10 mg QD	10 mg QD	10 mg QD	NR^a^	10 mg QD
Dextromethorphan	30 mg QD	30 mg QD	30 mg BID (↑)^b^	NR^a^	30 mg BID
Piracetam	600 mg QD	1200 mg QD (↑)	600 mg BID (Δ)^c^	NR^a^	600 mg BID
Risperidone	0.5 mg QD	0.5 mg QD	0.5 mg QD	NR^a^	D/C^d^
Aripiprazole	—	—	2.5 mg QD (★)	NR^a^	2.5 mg QD
Alprazolam	—	—	—	—	0.25 mg PRN (★)

Follow-up course

At the December 2025 follow-up, the patient reported improved mood and better work performance. She described fewer dissociative episodes and fewer unnoticed mistakes. She also reported morning tiredness. Because some symptoms remained and there was a goal of further supporting cognitive integration, piracetam was increased to 1200 mg daily, while the other medications were continued unchanged.

By January 2026, she described herself as more stable overall and stated that she was no longer experiencing blanking-out episodes. At this point, dextromethorphan was increased to 60 mg daily and administered as 30 mg twice daily. Piracetam was continued at 1200 mg daily, but was also divided into twice-daily dosing as 600 mg twice daily. Aripiprazole 2.5 mg once daily was added, while risperidone 0.5 mg daily was maintained.

In March 2026, the PHQ-9 score had fallen to 6, consistent with mild depressive symptoms, while the GAD-7 score was 14, still reflecting moderate anxiety. She reported residual concentration problems and occasional guilt or self-critical thoughts. She denied medication side effects. No further changes were made to the core regimen at that visit. No prescription list was included in the 9 March 2026 note.

At the 4 May 2026 follow-up, she reported that her mood remained stable and that her work performance had continued to improve. She stated that she was no longer experiencing dissociative fugue-like episodes. Risperidone had been discontinued, as she was no longer taking it. Aripiprazole 2.5 mg daily, dextromethorphan 60 mg daily in split dosing, piracetam 1200 mg daily in split dosing, and fluoxetine 10 mg daily were continued. Low-dose alprazolam 0.25 mg was prescribed as needed for acute emotional distress related to relationship conflicts. She also noted a reduction in appetite while taking aripiprazole. The clinical note did not state whether this was experienced as beneficial or adverse, and causality could not be determined.

No trauma-focused psychotherapy referral, psychotherapy start date, session frequency, session duration, or psychotherapy response was documented.

Key clinical observations

Over the documented follow-up period, the patient showed progressive resolution of dissociative and fugue-like symptoms. Sudden unresponsiveness, blanking-out episodes, and memory gaps ceased and remained absent by the final follow-up. This improvement occurred alongside better occupational accuracy and improved self-monitoring at work. Morning tiredness was reported early in treatment, but the available record does not allow firm attribution to any specific medication or dosing time. Generalized anxiety and emotional reactivity triggered by interpersonal stress persisted, although later use of as-needed alprazolam was intended to manage acute distress.

## Discussion

Diagnostic formulation

The working diagnosis was trauma-related dissociation with chronic derealisation, recurrent blank-outs or fugue-like episodes, and comorbid depressive and anxiety symptoms. The patient’s derealisation, abrupt unresponsiveness, memory disruption, and occupational self-monitoring failures were clinically consistent with dissociative phenomena as defined in DSM-5-TR, but the available record did not document a structured dissociation interview or a validated dissociation scale [1,17].

This case does not formally establish dissociative PTSD. Dissociative PTSD requires that full PTSD criteria are met, together with persistent or recurrent derealisation, depersonalisation, or both [1-4]. The patient had trauma-related guilt and intrusive imagery associated with her paternal grandfather’s sudden death, but the record did not document a complete PTSD criterion review, including Criterion A trauma qualification, avoidance, negative cognition/mood symptoms, arousal symptoms, duration, impairment, and exclusion criteria. Therefore, the most accurate wording is trauma-related dissociation rather than confirmed dissociative PTSD.

Trajectory and durability of response

The longitudinal course showed gradual and sustained improvement rather than a dramatic early response followed by a plateau. Depressive symptoms, measured by the PHQ-9, declined from the moderate range at baseline to the mild range by March 2026. The most meaningful improvements, however, were functional. The patient made fewer errors at work, became more able to monitor her own performance, and stopped experiencing blank-outs and fugue-like episodes. These gains were maintained through the 4 May 2026 visit, suggesting that the benefits consolidated over time rather than representing a brief or unstable response.

Specificity of benefit

The clearest benefit was seen in dissociative and fugue-like phenomena, including abrupt memory gaps, sudden unresponsiveness, and trauma-linked disruptions in cognitive integration. These symptoms resolved progressively and remained absent later in treatment. By contrast, generalized anxiety and relationship-triggered emotional reactivity showed only partial improvement and were less stable. This pattern is clinically important because it helps distinguish the presentation from primary attention-deficit/hyperactivity disorder or uncomplicated anxiety. In those conditions, such a selective and sustained improvement in dissociative and executive symptoms would be less expected, although formal neuropsychological testing was not performed, and objective executive-function improvement cannot be claimed.

Dose and schedule considerations

Medication dose and frequency appeared clinically relevant. Dextromethorphan was increased from 30 mg/day to 60 mg/day in January 2026, and both dextromethorphan and piracetam were divided into twice-daily dosing. The patient then reported better daytime stability, and blanking-out episodes were no longer reported. Because several changes occurred at the same time, no single adjustment can be identified as responsible. Still, the course is consistent with the possibility that more sustained daytime glutamatergic activity supported better cognitive continuity during work demands and interpersonal stress. This interpretation remains speculative because medication timing, adherence patterns, alcohol changes, and objective cognitive measures were incompletely documented.

Mechanistic interpretation

The clinical course is broadly consistent with emerging models of developmental synaptic vulnerability in PTSD. Recent analyses have emphasized pathways involved in synaptic pruning, complement signalling, and oligodendrocyte maturation, suggesting that early mis-pruning may create fragile corticolimbic and white-matter circuits that become overwhelmed after trauma exposure [7]. However, this pruning-vulnerability cascade remains a proposed theoretical framework rather than an established diagnostic or treatment model.

In this case, progressive improvement in dissociative and executive symptoms after intensification of NMDA-AMPA modulation may reflect reopening of plasticity pathways in circuits affected by developmental and trauma-related dysregulation. Potential mechanisms include BDNF and mTOR signalling, which have been linked to AMPA-dependent effects in ketamine models [18,19]. The persistence of anxiety and interpersonal reactivity also fits the broader pruning-vulnerability cascade model, in which later-stage senescence, attachment-related processes, and systemic symptom consolidation may continue even after partial restoration of plasticity [7,8]. An alternative explanation is that improvement reflected nonspecific clinical support, natural symptom fluctuation, reduced alcohol intake, placebo effects, antidepressant response, antipsychotic effects, or occupational adaptation rather than glutamatergic augmentation alone.

Medication evolution, safety, and practical considerations

Dextromethorphan is an NMDA-receptor antagonist and sigma-1 receptor agonist that is rapidly metabolised through CYP2D6; in approved dextromethorphan-bupropion treatment for major depressive disorder, CYP2D6 inhibition is used to increase dextromethorphan exposure [13,14]. In this case, fluoxetine was used as the CYP2D6-inhibiting antidepressant. This use was off-label for dissociation. Potential precautions include serotonergic toxicity when combined with other serotonergic agents, misuse potential at high doses, dizziness, somnolence, and drug interactions.

Fluoxetine is a selective serotonin reuptake inhibitor and potent CYP2D6 inhibitor [20]. It may treat depressive and anxiety symptoms and may pharmacokinetically augment dextromethorphan exposure. Important precautions include serotonin syndrome, drug interactions, activation, gastrointestinal symptoms, sleep disturbance, sexual dysfunction, and suicidal ideation monitoring in young adults [15].

Piracetam is a racetam-class nootropic that has been reported to modulate neurotransmission, including glutamatergic and cholinergic systems, and to influence neuroplasticity, although its role in trauma-related dissociation is not established [21]. Its use here was off-label. Potential precautions include insomnia or somnolence, nervousness, gastrointestinal symptoms, bleeding-risk considerations in susceptible patients, and renal-dose considerations.

Risperidone and aripiprazole were not the primary glutamatergic agents but were part of the evolving regimen. Risperidone is a dopamine-serotonin antagonist used in psychotic disorders, bipolar disorder, and irritability associated with autism, with potential adverse effects including extrapyramidal symptoms, hyperprolactinaemia, sedation, metabolic effects, and QT-related precautions [22]. Aripiprazole is a dopamine-serotonin partial agonist/antagonist used in schizophrenia, bipolar disorder, and adjunctive major depressive disorder, with potential adverse effects including akathisia, insomnia, nausea, impulse-control problems, and metabolic monitoring needs [23]. In this case, low-dose risperidone appeared to provide early containment, but it was later discontinued without apparent loss of stability. Aripiprazole 2.5 mg once daily was continued.

Alprazolam 0.25 mg as needed was added only at the final documented visit for acute relationship-triggered distress. Benzodiazepines may reduce acute anxiety but are generally used cautiously in PTSD because of dependence, sedation, cognitive impairment, and potential interference with trauma-focused psychotherapy.

Overall tolerability was reassuring during the documented period. There were no reported serious adverse events, including serotonin toxicity, manic switching, worsening dissociation, or significant cardiovascular changes. However, vital signs, physical examination findings, and laboratory monitoring were not documented in the available record. These observations are consistent with earlier open-label reports suggesting that carefully titrated oral glutamatergic regimens can be feasible in outpatient settings [11,12], but they do not establish safety or efficacy.

Comparison with previous literature

This case differs from ketamine PTSD trials, which generally involve supervised intravenous administration and shorter acute-response windows [9,10]. It also differs from previously described oral glutamatergic case-series observations because the present report provides a longer longitudinal outpatient course with repeated PHQ-9 and GAD-7 measurements, medication-frequency changes, and functional occupational observations [11,12]. The main convergence is that NMDA-linked treatment approaches may improve trauma-related symptoms; the main difference is that this case showed the most prominent improvement in dissociative continuity and work functioning rather than complete remission of anxiety.

Compared with standard PTSD treatment literature, this case should not be interpreted as replacing trauma-focused psychotherapy or guideline-supported pharmacotherapy [5,6]. Rather, it suggests that glutamatergic augmentation may be a hypothesis-generating adjunctive strategy for selected patients with prominent dissociative and cognitive-integration symptoms.

Limitations

This report has several limitations. As a single case, it cannot establish causality. Multiple medication changes occurred during follow-up, making it difficult to determine the specific contribution of each agent. Alcohol use was also incompletely documented, which is important because changes in drinking could have affected both baseline symptoms and later improvement. No validated dissociation scale, structured PTSD interview, objective cognitive testing, formal mental status examination, physical or neurological examination, vital signs, laboratory tests, or radiological investigations were documented. PHQ-9 and GAD-7 scores were also missing at the final 4 May 2026 visit. In addition, anxiety and interpersonal difficulties persisted, indicating that the regimen did not address all parts of the patient’s presentation. This supports the likely need for adjunctive trauma-focused, grief-oriented, or phase-oriented psychotherapy during periods of improved stability, although such psychotherapy was not documented as part of this case.

Clinical and research implications

**Figure 1:**
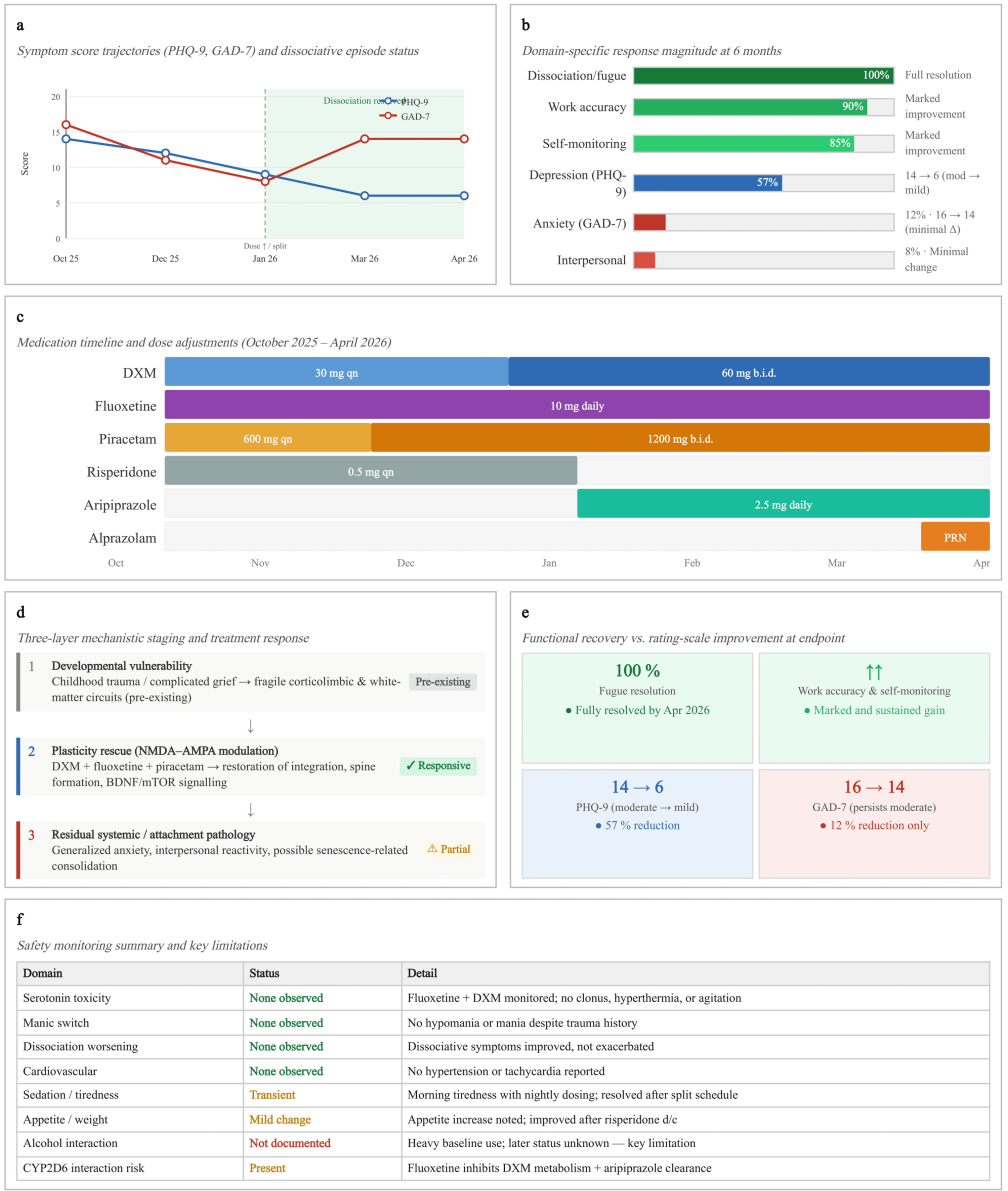
Longitudinal clinical course of a 25-year-old woman with childhood trauma, complicated grief, chronic derealisation, and recurrent fugue-like episodes treated with an oral NMDA-AMPA-focused regimen BDNF, brain-derived neurotrophic factor; DXM, dextromethorphan; GAD-7, Generalised Anxiety Disorder-7; mTOR, mammalian target of rapamycin; PHQ-9, Patient Health Questionnaire-9; PRN, as needed This figure presents the six-month longitudinal outcomes of oral NMDA-AMPA augmentation in trauma-related dissociation: symptom trajectories, domain specificity, medication evolution, mechanistic staging, functional recovery, and safety profile. (a) PHQ-9 (blue) and GAD-7 (red) scores across five visits; green shading indicates the period of dissociative symptom resolution. The dashed vertical line marks dose intensification and split dosing (January 2026). (b) Domain-specific response magnitude at six months, showing the strongest benefit in dissociative/fugue phenomena and occupational function, with partial or minimal response in anxiety and interpersonal reactivity. (c) Medication timeline illustrating the evolution from once-nightly dosing to twice-daily split regimen, risperidone-to-aripiprazole transition, and PRN alprazolam addition. (d) Three-layer mechanistic staging derived from the pruning-vulnerability cascade model: developmental vulnerability (Layer 1, pre-existing), plasticity rescue via NMDA-AMPA modulation (Layer 2, targeted and responsive), and residual systemic/attachment pathology (Layer 3, partially responsive). (e) Dissociation between functional recovery (occupational accuracy, self-monitoring, fugue resolution) and residual rating-scale elevation, emphasising that functional outcomes may better capture the treatment effect. (f) Safety monitoring outcomes and key study limitations, including alcohol use as an unmeasured confounder. Image credit: Ngo Cheung. Figure created using Microsoft PowerPoint (Microsoft® Corp., Redmond, WA).

This case adds to the growing clinical literature suggesting that oral NMDA-AMPA-focused regimens can be implemented in routine outpatient care for trauma-related dissociation and executive dysfunction. It also highlights the importance of tracking functional outcomes, such as work accuracy and self-monitoring, alongside symptom scales (Figure 1). Dose frequency may also matter, particularly when daytime cognitive continuity remains impaired.

Future studies should use prospective controlled designs; structured diagnostic interviews; validated PTSD and dissociation scales, such as the Clinician-Administered PTSD Scale and the Dissociative Subtype of PTSD Scale; objective cognitive testing; systematic alcohol and substance-use monitoring; adverse-event checklists; vital-sign and laboratory monitoring; and predefined medication algorithms. Research should also compare oral glutamatergic augmentation alone with combined models that include grounding skills, stabilisation, trauma-focused psychotherapy, grief-focused therapy, or phase-oriented treatment for dissociative presentations.

## Conclusions

Over the documented outpatient follow-up period, an oral NMDA-AMPA-focused regimen was associated with progressive and durable remission of chronic derealisation, fugue-like episodes, and occupational impairment in a young woman with trauma-related dissociation. The most striking improvement was restoration of cognitive integration and work performance, especially after regimen intensification and split dosing. Residual generalized anxiety and interpersonal reactivity persisted, suggesting that neuroplasticity-focused medication may address only one layer of trauma-related pathology. As a single case with polypharmacy, incomplete diagnostic measurement, and missing final rating scales, this report should be interpreted as hypothesis-generating rather than causal evidence. Further controlled research is needed, including studies that integrate glutamatergic augmentation with structured trauma-focused or phase-oriented psychotherapy.
